# Investigation of the Prevalence of Associated Genetic Mutations (Co-Mutations) in Patients with Actionable Driver Mutations in Lung Cancer: A Retrospective Study

**DOI:** 10.3390/diagnostics16071106

**Published:** 2026-04-07

**Authors:** Abed Agbarya, Walid Shalata, Edmond Sabo, Leonard Saiegh, Yuval Shaham, Haitam Nasrallah, Kamel Mhameed, Salam Mazareb, Mohammad Sheikh-Ahmad, Dan Levy Faber

**Affiliations:** 1 The Ruth and Bruce Rappaport Faculty of Medicine, Technion-Israel Institute of Technology, 1 Efron Street, Haifa 3109601, Israel; shahamyuval8@gmail.com (Y.S.); mohammad.ahmad@b-zion.org.il (M.S.-A.); danfaber1@hotmail.com (D.L.F.); 2 Department of Oncology, Bnai-Zion Medical Center, 47 Eliyahu Golomb Avenue, Haifa 3339419, Israel; 3 The Legacy Heritage Cancer Center, Dr. Larry Norton Institute, Soroka Medical Center, 151 Yitzhack Rager Avenue, Beer Sheva 8457108, Israel; walid.shalata@gmail.com; 4 Faculty of Health Sciences, Ben Gurion University of the Negev, 1 Ben Gurion Avenue, Beer Sheva 8410501, Israel; 5 Pathology Institute, Lady Davis Carmel Medical Center, 7 Michal Street, Haifa 3436212, Israel; edmondsa@clalit.org.il (E.S.); mazareb_salam@clalit.org.il (S.M.); 6 Institute of Endocrinology, Bnai-Zion Medical Center, 47 Eliyahu Golomb Avenue, Haifa 3339419, Israel; leonard.saiegh@gmail.com; 7 The Joseph Fishman Oncology Center, Rambam Medical Center, 6 Efron Street, Haifa 3525409, Israel; h_nasrallah@rmc.gov.il (H.N.); k_mhameed@rmc.gov.il (K.M.); 8 Department of Cardiothoracic Surgery, Lady Davis Carmel Medical Center, 7 Michal Street, Haifa 3436212, Israel

**Keywords:** lung cancer, co-mutations, genetic mutations, actionable mutations, driver mutations, genomic cluster

## Abstract

**Background/Objectives**: Lung cancer remains the leading cause of cancer-related mortality globally. Approximately 45% of these tumors harbor oncogenic mutations that drive carcinogenesis and are amenable to targeted therapies. Other predictive biomarkers—e.g., PD-L1, TMB, and MSI—play a crucial role in patients’ management. This study aims to investigate the existence of mutation clusters (co-mutations) and evaluate the correlation of these clusters with various clinical and laboratory parameters. **Methods**: A retrospective study was conducted utilizing pathological samples from lung cancer patients harboring mutations in *EGFR*, *KRAS*, *ALK*, *BRAF*, *MET*, *HER2*, *ROS1*, *NTRK*, and *NRG1*. Data were collected from the Institute of Pathology at Carmel Medical Center between the years 2022 and 2024. Patients were stratified using a Two-Step Cluster Analysis algorithm based on actionable mutations and co-mutations. Heatmaps and dendrograms were generated to assess the correlation between these genomic clusters, clinical metrics, and predictive biomarkers. **Results**: The study cohort included 129 patients with actionable mutations. Five distinct clusters were identified: Clusters 1, 2, and 3 exhibited a high expression of *STK11* and *TP53* co-mutations alongside *KRAS* drivers (*n* = 38, *n* = 12, and *n* = 23, respectively). Clusters 4 and 5 demonstrated high expression of *ALK* alterations and tumor suppressor gene mutations (*n* = 31 and *n* = 25, respectively). Cluster comparisons demonstrated statistically significant differences between clusters regarding age, gender, PD-L1 expression, and tumor mutational burden. No significant associations were found regarding ethnicity or microsatellite instability status. **Conclusions**: By constructing clusters based on the aggregate of genomic alterations in patients with actionable mutations, it is possible to predict associations with distinct demographic and clinical characteristics. Future research should apply this analytical approach to larger cohorts to further characterize these subgroups and investigate potential correlations with therapeutic efficacy.

## 1. Introduction

Lung cancer is a primary cause of cancer mortality worldwide, resulting in high death rates among both men and women [1,2,3]. Despite increasing awareness regarding early diagnosis, lung cancer is frequently detected at advanced stages where therapeutic options are limited, resulting in a poor prognosis [1]. Consequently, frequent screening of high-risk individuals is essential to facilitate early detection and significantly improve survival rates. The accurate and early diagnosis of pulmonary lesions is critical to tailoring the most appropriate therapeutic regimen and improving patient outcomes [1].

Lung cancer is defined as the uncontrolled growth of abnormal cells originating in the lungs, leading to tumor formation and compromised pulmonary function. It originates in the epithelium of the large and small bronchi and may metastasize via the lymphatic system or hematogenous spread, with common metastatic sites including the liver, adrenal glands, bone, and the central nervous system [1,4,5].

The incidence of lung cancer is steadily rising. In 2020, it was the second most diagnosed cancer (following breast cancer), accounting for 11.4% of 19.3 million cases. By 2022, it became the most diagnosed malignancy, with nearly 2.5 million new cases, representing 12.4% of all global cancer cases (one in eight new cancers). Regarding mortality, lung cancer is the leading cause of cancer death, rising from 18% in 2020 to 18.7% in 2022 [2,3]. The primary risk factor is tobacco smoking, though non-smokers exposed to second-hand smoke are also at risk. Additional risk factors include occupational exposure to carcinogens (e.g., asbestos, radon), air pollution, chronic lung diseases (e.g., COPD), and hereditary cancer syndromes [1].

Lung malignancies are classified into two main histological groups: Non-Small Cell Lung Carcinoma (NSCLC) and Small Cell Lung Carcinoma (SCLC). NSCLC accounts for 80% of cases, while SCLC comprises nearly 20%. This study focuses on NSCLC, which is further subdivided into histological subtypes, primarily Squamous Cell Carcinoma (SCC) and Adenocarcinoma (ADC), each accounting for 30–40% of cases. Large Cell Carcinoma (LCC) is a less common subtype, representing just under 10% of cases [5].

Current diagnostic and therapeutic strategies for lung cancer rely on histological diagnosis and molecular profiling [6]. Advanced next-generation sequencing (NGS) allows for the high-quality detection of a broad spectrum of mutations [7]. A significant proportion of NSCLC tumors harbor actionable mutations—genetic alterations treatable with targeted biological therapies rather than cytotoxic chemotherapy. The most prevalent actionable mutations discussed herein include Epidermal Growth Factor Receptor (*EGFR*), *Kirsten rat sarcoma virus (KRAS) G12C*, and *ALK* [8,9,10,11].

The *KRAS G12C* mutation is the most common alteration in the *KRAS* gene, which encodes a key protein in cell proliferation pathways. *KRAS* mutations are present in 15–20% of NSCLC cases. This specific mutation is targetable by two biological agents, sotorasib and adagrasib, which selectively bind covalently to the KRAS protein, inhibiting its activity [12,13,14].

*EGFR* (Epidermal Growth Factor Receptor) mutations occur in approximately 15% of NSCLC cases in Western Europe and the USA and are frequently associated with non-smokers. *EGFR* encodes a transmembrane tyrosine kinase receptor involved in cell proliferation cascades. These mutations are treated with Tyrosine Kinase Inhibitors (TKIs) such as Osimertinib [8,10,11,14,15].

*ALK* (Anaplastic Lymphoma Kinase) gene rearrangements result from translocation, typically with *EML4*. Found in 3–7% of NSCLC cases, this translocation leads to the overexpression of the ALK tyrosine kinase receptor, driving cell growth. This alteration is managed with TKIs such as crizotinib, ceritinib, lorlatinib, and alectinib [16]. Additional, less common actionable mutations include *BRAF*, *MET*, *HER2*, *RET*, *ROS1*, *NTRK*, and *NRG1* [11,17].

Beyond molecular profiling, immunotherapeutic biomarkers such as PD-L1 expression, tumor mutational burden (TMB), and microsatellite instability (MSI) aid in treatment selection for NSCLC [18,19,20]. PD-L1 (Programmed Death-Ligand 1) expression on tumor cells suppresses the immune response by interacting with PD-1 on T-cells, inhibiting effector T-cells and promoting regulatory T-cells. Immune Checkpoint Inhibitors (ICIs) block this pathway to restore anti-tumor immunity [18]. TMB quantifies mutations within tumor DNA; high TMB correlates with better response to immunotherapy [19]. MSI refers to hypermutable DNA microsatellites due to defective mismatch repair (MMR) mechanisms and serves as a predictive marker for immunotherapy response [20].

This study utilized statistical analysis to determine whether specific clusters of co-mutations accompany actionable driver mutations. We specifically examined significant co-mutations including *TP53*, *KRAS*, *STK11*, *KEAP1*, and *CDKN2A*, alongside others of unclear significance. The existence of such clusters may influence prognosis and the efficacy of targeted therapies. We hypothesized that such clusters exist and aimed to identify them to optimize future therapeutic strategies. Additionally, we assessed the correlation of these clusters with biomarkers (PD-L1, TMB, MSI) and demographic parameters (age, gender, ethnicity) to identify patterns that may inform future studies on clinical efficacy and toxicity.

## 2. Materials and Methods

### 2.1. Study Design and Population

This retrospective, non-interventional study included 129 patient samples archived at the Institute of Pathology database at Carmel Medical Center, Haifa. The cohort consisted of patients diagnosed with NSCLC who possessed a complete molecular profile containing at least one of the following actionable mutations: *EGFR*, *KRAS*, *ALK*, *BRAF*, *MET*, *HER2*, *ROS1*, *NTRK*, or *NRG1*. Lung tumor biopsy samples collected between 2022 and 2024 were included. Patients were screened based on the presence of specific actionable mutations defined in the inclusion criteria.

### 2.2. Data Collection

Following the initial screening of 129 patients, relevant data were extracted using pathological reports. Subsequently, statistical analysis and result interpretation were performed. All samples were sourced from the existing repository at the Institute of Pathology, Carmel Medical Center. Following data collection and screening, statistical analysis was performed on an anonymized dataset (identified only by serial numbers).

### 2.3. Molecular Profiling

All molecular profiling was performed on formalin-fixed paraffin-embedded tumor specimens as part of routine clinical testing at Carmel Medical Center Pathology Department laboratories. Next-generation sequencing was performed using the Oncomine™ Comprehensive Assay v3 (Thermo Fisher Scientific, Waltham, MA, USA), which covers 161 genes encompassing major cancer drivers and tumor suppressors. The Oncomine Dx Target Test detects 46 cancer driver gene variants, including *EGFR* mutations (including L858R, T790M, and exon 19 deletions); *BRAF*, *KRAS*, *ERBB2*, and *MET* exon 14 skipping mutations; and *ALK*, *ROS1*, *RET*, and *NTRK1/2/3* fusions. The assay is validated to detect single-nucleotide variants and small insertions/deletions, and to detect gene fusions and copy number alterations.

Tumor mutational burden (TMB) was defined as the number of somatic mutations per megabase (mut/Mb) within the panel’s reportable coding territory.

Microsatellite instability (MSI) was assessed using MSI status, reported as MSI-high versus microsatellite-stable (MSS).

### 2.4. Ethics

The study was approved by the Helsinki Committee of Carmel Medical Center protocol CMC-0073-24 on 23 September 2024.

### 2.5. Statistical Analysis

Cluster analysis was performed based on actionable mutations, co-mutations, and copy number variations (CNVs) using a Two-Step Cluster Analysis algorithm. This hybrid method is designed for categorical and/or continuous data and operates in two phases.

#### 2.5.1. Pre-Clustering

The algorithm scans data to create small sub-clusters using a log-likelihood distance measure suitable for categorical (binary) variables. Since our variables are binary (presence or absence of a mutation), a log-likelihood distance metric estimates similarity based on the likelihood that data points will appear in the same cluster.

#### 2.5.2. Hierarchical Clustering

Sub-clusters are merged into final clusters based on model fit criteria (BIC or AIC). The algorithm determines the optimal number of clusters automatically, though manual specification is possible.

Five clusters were generated for all patients. Heatmaps were created for visualization using ArrayGen (Undri, Pune, Maharashtra, India). Heatmaps visually represent the relationships between mutation types across patients. The color gradient ranges from light yellow (0; absence/low presence of a mutation) to dark brown (+2; high intensity/prevalence of the mutation). It is important to note that in binary data clustering, color intensity reflects the prevalence or significance of a mutation within the context of the cluster. Heatmaps depict binary alteration status per patient (0 = absent; 1 = present). Color intensity is used solely as a visual encoding of binary status and does not represent continuous measurements. If aggregated heatmaps are displayed (by cluster), the color scale reflects the proportion of cases within the cluster harboring the alteration (0–100%).

#### 2.5.3. Dendrograms

A dendrogram was constructed for each heatmap to visualize the hierarchical structure. It illustrates the similarity between patient mutation profiles or the co-occurrence of specific mutations. Branch height indicates the degree of dissimilarity; shorter distances imply high similarity.

#### 2.5.4. Comparative Statistics

Clusters were compared based on demographics (age, gender, ethnicity) and biomarkers (PD-L1 through immunohistochemistry, TMB, and MSI/MSS via NGS). Continuous variables were tested for normality using the Kolmogorov–Smirnov test and analyzed using Student’s *t*-test or Mann–Whitney U test as appropriate. Bonferroni correction was applied to address multiple hypothesis testing. Categorical variables were analyzed using Chi-square or Fisher’s exact tests. A two-tailed *p*-value of ≤0.05 was considered statistically significant. Analysis was performed using IBM SPSS version 26 (IBM, Armonk, NY, USA).

Clustering was performed on a binary matrix indicating the presence (1) or absence (0) of each genomic alteration per case. The clustering inputs included (1) actionable driver alterations (*EGFR*, *KRAS*, *ALK*, *BRAF*, *MET*, *HER2*, *ROS1*, *NTRK*, *NRG1*) and (2) co-occurring alterations and copy number changes selected a priori based on biological relevance and/or prevalence in the cohort (e.g., *TP53*, *STK11*, *KEAP1*, *CDKN2A*, *ARID1A*).

Two-step clustering was performed using the log-likelihood distance for categorial/binary inputs with the two-step procedure’s standard pre-clustering stage followed by hierarchical agglomeration. The optimal number of clusters was selected by BIC/AIC as implemented in the software, and we report the resulting cluster quality using the silhouette coefficient (average silhouette width). To assess stability, we performed a sensitivity analysis by reporting the concordance of cluster assignments across resamples using the adjusted Rand index, and confirming that the major cluster-defining patterns were preserved.

Continuous variables (e.g., age, PD-L1 percentage, TMB, mut/Mb) were summarized as mean ± SD or median (IQR), depending on distribution; categorial variables were summarized as counts and percentage. For comparisons across the five clusters, we used an omnibus test first (one-way ANOVA for approximately normal variables or Kruskal–Wallis for non-normal variables), followed by post hoc pairwise comparisons only when the omnibus test was significant. We controlled multiplicity using Bonferroni correction within each prespecified family of pairwise comparisons and report both unadjusted and Bonferroni-adjusted *p*-values. Alongside *p*-values, we report effect sizes with 95% confidence intervals for continuous outcomes (Cohen’s d) and for categorical outcomes odds ratios/risk differences as applicable.

## 3. Results

To analyze binary categorical data (presence/absence of mutations), a Two-Step Cluster Analysis was performed on 129 patients’ samples. This unsupervised algorithm grouped patients based on mathematical proximity. Five clusters were generated (cluster 1, *n* = 38; cluster 2, *n* = 12; cluster 3, *n* = 23; cluster 4, *n* = 31; and cluster 5, *n* = 25).

Each cluster was visualized using a dendrogram and a heatmap. In the heatmaps, mutations (*x*-axis) were color-coded to distinguish between primary mutations, co-mutations, and CNVs. Patients (*y*-axis) were listed by serial number. AI tools (Cloud, ChatGPT 5.2 deep research) were utilized to help identify mutation patterns within the algorithmic clusters.

### 3.1. Clustering of Actionable Mutation and Co-Mutation Cluster Patterns

#### 3.1.1. Cluster 1 (*n* = 38)

**Figure 1:**
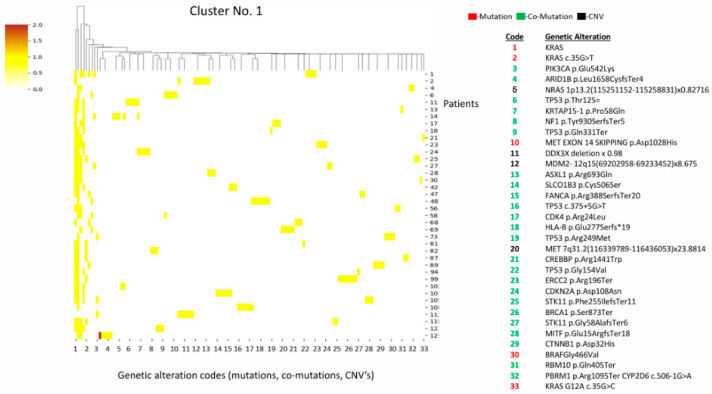
Heatmap and cluster 1 pattern. Key alterations: *KRAS* mutations (multiple, including G12A, G12A, G12D, G12V); STK11 mutations; TP53 mutations (several loss-of-function); *MET* exon 14 skipping + *MET* amplification; *MDM2* amplification; *NF1* and *PIK3CA* mutations. Pattern: high frequency of *TP53* mutations; frequent *KRAS/STK11* co-mutations; *MET* alterations (exon 14 skipping + amplification).

Cluster 1 is characterized by *KRAS* mutations (various subtypes) alongside *STK11* co-mutations, a high frequency of *TP53* mutations, and a high concentration of *MET* alterations (exon 14 skipping + amplification) (Figure 1).

#### 3.1.2. Cluster 2 (*n* = 12)

**Figure 2:**
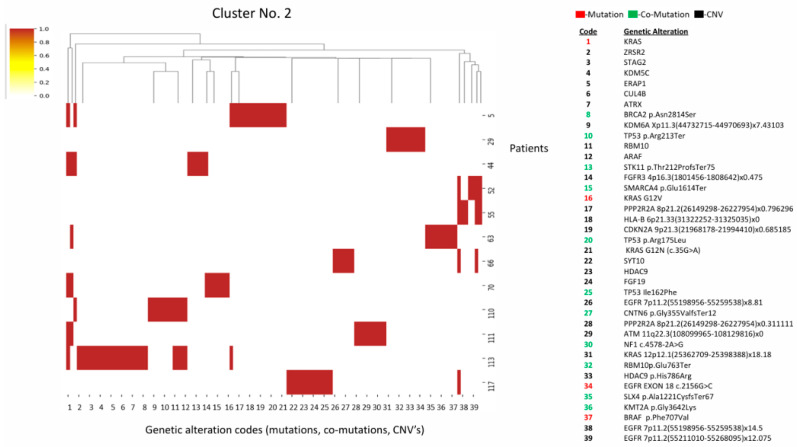
Heatmap and cluster 2 pattern. Key alterations: *KRAS* mutations (G12V, G12N); *STK11* mutations; *EGFR* amplification (CNV), *STK11*, *TP53* mutations, multiple chromatin modifiers: *KDM6A*, *ATRX*, *SMARCA4*; HLA-B deletion. Pattern: *KRAS/STK11/TP53* triad similar to cluster 1, but with more CNVs; *EGFR* CNV amplifications; chromatin remodeling gene alterations.

Cluster two has a similar *KRAS/STK11/TP53* triad to cluster 1, but is distinguished by a higher frequency of *EGFR* CNV amplifications and mutations in chromatin remodeling genes (*KDM6A*, *ATRX*, *SMARCA4*) (Figure 2).

#### 3.1.3. Cluster 3 (*n* = 23)

**Figure 3:**
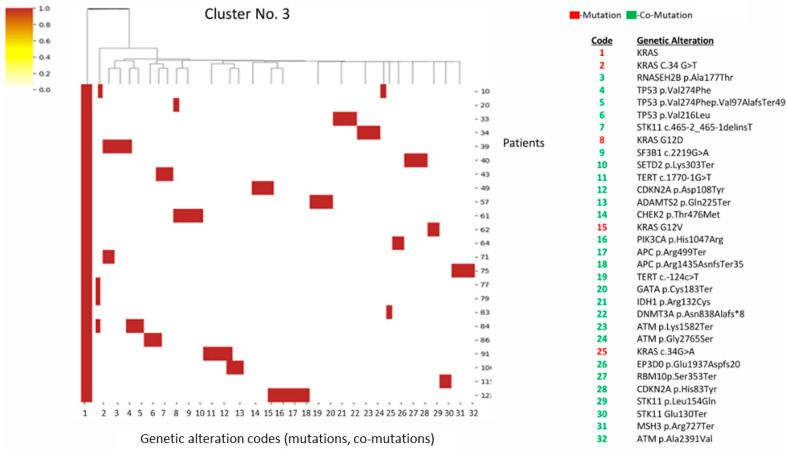
Heatmap and cluster 3 pattern. Key alterations: *KRAS* mutations (G12D, G12V, c.34G>A); co-occurring *STK11* mutations; *TP53*, *CDKN2A*, *ATM*, *TERT*, *SETD2*, and *APC*. Pattern: Strong presence of *KRAS + STK11* mutations, but less *TP53* activity than cluster 1; presence of DNA repair pathway genes: *ATM*, *CHEK2*, *DNMT3A*, *IDH1*.

Cluster 3 has a high presence of *KRAS* and *STK11* co-mutations, but notably lower *TP53* frequency compared to clusters 1 and 2. Contains mutations in DNA repair pathways (Figure 3).

#### 3.1.4. Cluster 4 (*n* = 31)

**Figure 4:**
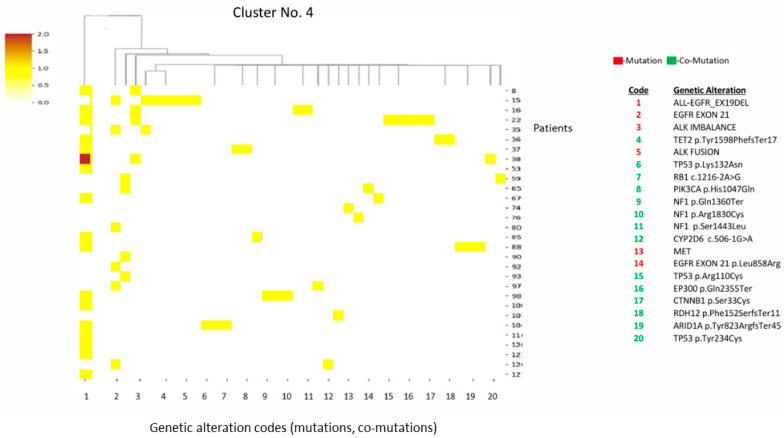
Heatmap and cluster 4 pattern. Key alterations: *EGFR*-activating mutations (EX19DEL, EX21 L858R); *ALK* fusions; *TP53*, *ARID1A*, *PIK3CA*. Pattern: *EGFR/ALK* oncogene-driven tumors; less genomic instability, more oncogene-addicted.

Cluster 4 is defined by *EGFR*-activating mutations co-occurring with *ALK* fusions. This represents an oncogene-driven profile with less genomic instability (Figure 4). Within cluster 4, *ALK* fusions were identified by NGS, performed as part of routine diagnostics. The biopsy timing for all cases was obtained at initial diagnosis prior to exposure to *EGFR*- or *ALK*-targeted therapy. These details are provided to support interpretation given that *EGFR* and *ALK* alterations are typically mutually exclusive on treatment-naïve NSCLC, although rare concomitant cases have been reported.

#### 3.1.5. Cluster 5 (*n* = 25)

**Figure 5:**
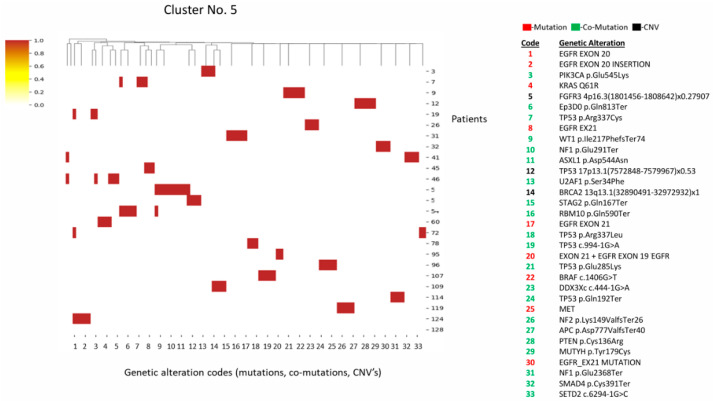
Heatmap and cluster 5 pattern. Key alterations: *EGFR* exon 20 mutations/insertions; *NF1*, *NF2*, *TP53*, *PTEN*, *SETD2*; *PIK3CA*, *BRAF*, *KRAS* (Q61R); similar to cluster 4, but with more diverse tumor suppressor loss. Pattern: *EGFR* exon 20 mutations; tumor suppressor mutations.

Cluster 5 is characterized by *EGFR* exon 20 mutations/insertions. Unlike cluster 4, these tend to co-occur with tumor suppressor loss (e.g., *NF1*, *TP53*, *PTEN*) (Figure 5).

### 3.2. Immunotherapeutic Biomarker Analysis

#### 3.2.1. PD-L1 Expression

**Figure 6:**
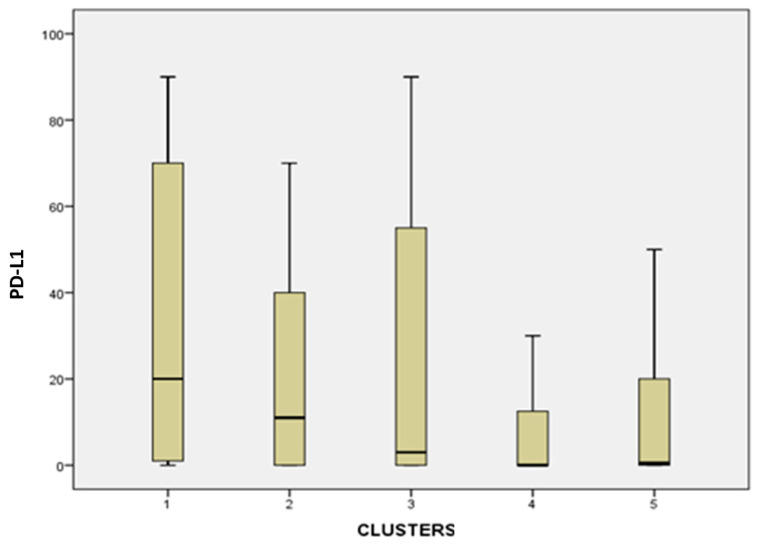
Boxplot graph of PD-L1 expression across the different clusters. The analysis was performed on a continuous variable with values ranging from 0 to 100. Between cluster 1 and 4, there is a difference with a significance level of *p* = 0.001. Between cluster 1 and 5, there is a difference with a significance level of *p* = 0.011. Between cluster 3 and 4, there is a difference with a significance level of *p* = 0.032. Between cluster 2 and 4, there is a non-significant difference, *p* = 0.061.

A comparison of clusters according to the ordinal categorical variable showed that the mean PD-L1 expression was lowest in cluster 4 samples (Figure 6). Cluster 4 had significantly lower PD-L1 expression compared to cluster 1 (*p* = 0.001) and cluster 3 (*p* = 0.032). Between cluster 1 and 5, there was a difference with a significance level of 0.011. Between cluster 3 and 4, there was a difference with a significance level of 0.032 (Figure 6).

#### 3.2.2. Tumor Mutational Burden (TMB)

**Figure 7:**
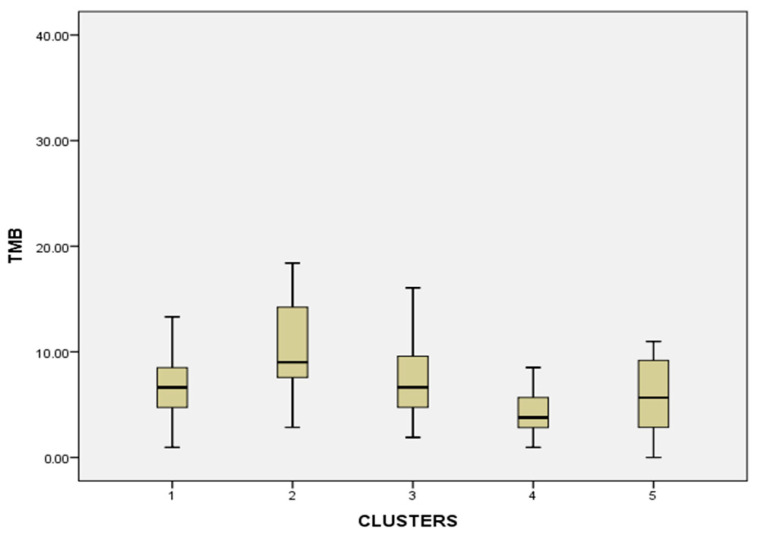
Boxplot graph of tumor mutational burden (TMB) expression across the different clusters. The analysis was conducted on a continuous variable.

TMB statistical significance: Cluster 4 had significantly lower TMB than clusters 1 (*p* = 0.012), 2 (*p* = 0.001), and 3 (*p* = 0.009). Cluster 2 had significantly higher TMB than clusters 1 (*p* = 0.013), 4 (*p* = 0.001), and 5 (*p* = 0.007) (Figure 7).

#### 3.2.3. MSI/MSS

No statistically significant differences were found between clusters in terms of their microsatellite instability; all evaluable patients were microsatellite-stable (MSS).

### 3.3. Demographic Analysis

#### 3.3.1. Age

**Figure 8:**
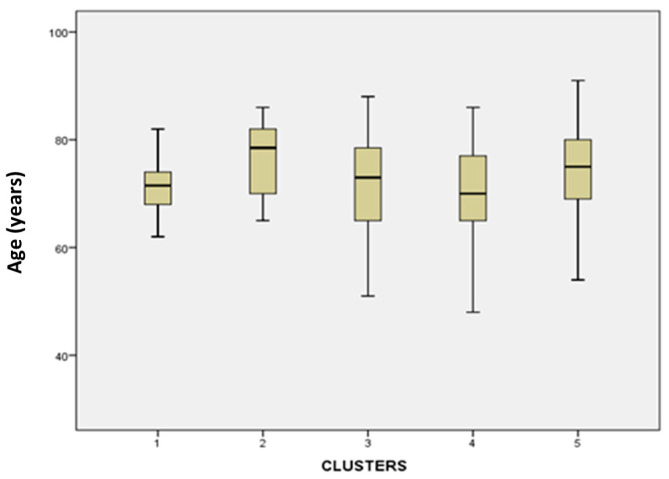
Boxplot graph of the age (years) variable across the different clusters.

Age of patients analysis (Figure 8) yielded significant differences were between cluster 1 vs. 2 (*p* = 0.026), cluster 1 vs. 5 (*p* = 0.031), cluster 2 vs. 4 (*p* = 0.039), and cluster 4 vs. 5 (*p* = 0.044). Clusters 1 and 4 represented a younger demographic population.

#### 3.3.2. Gender

**Figure 9:**
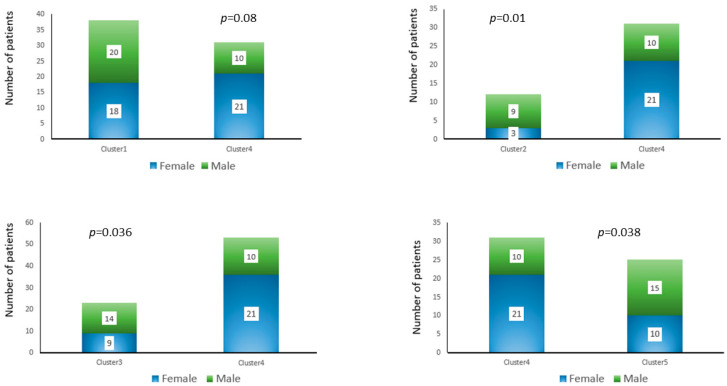
Comparison between genders among the clusters.

Figure 9 presents the comparison between genders among the clusters. Cluster 4 comprised significantly more women than men, while clusters 1, 2, 3, and 5 had fewer women than men. The distribution of men and women between cluster 1 and cluster 4 was not statistically significant. The findings of the analysis showed a statistically significant difference between cluster 2 and cluster 4 (*p* = 0.01), as well as between cluster 3 and cluster 4 (*p* = 0.036) and between cluster 4 and cluster 5 (*p* = 0.036).

#### 3.3.3. Ethnic

No significant differences were observed between Jewish and Arab patients’ ethnicity across clusters.

## 4. Discussion

This study sought to identify prevalent associations between co-mutations and actionable driver mutations in lung cancer patients, and to examine their correlations with immunotherapeutic biomarkers and demographics. Our goal was to establish a foundation for future research investigating the clinical implications of these genomic subgroups to enhance precision medicine in lung cancer and to contribute to improve the prognosis of lung cancer patients.

The cluster analysis revealed distinct genomic patterns. *KRAS*-driven tumors (clusters 1 and 3) frequently co-occurred with *STK11*. This finding aligns with recent publications on the biomarker *STK11*’s co-mutation with *KRAS* and the implication as per therapeutic outcome with neoadjuvant treatment using ICIs (immune checkpoint inhibitors) for NSCLC patients [21,22,23]. Furthermore, clusters 1 and 3 exhibited varying degrees of *TP53* involvement, which is in line with Schabath’s 2015 study [24]. Skoulidis et al. [25] observed that, in lung adenocarcinoma, PD-L1 expression plays a role in the response of *STK*11 and *TP53* co-mutations with *KRAS*. In the current study, cluster 1 displayed high PD-L1 expression, suggesting potential immunogenicity. Suzawa et al.’s [26] findings indicated that *KRAS* mutation is common in NSCLC tumors harboring *MET* alteration. Similarly, in the present study, cluster 1 was associated with high levels of *MET* alterations. These co-existing genomic changes could each be targeted by specific agents; however, due to reported therapeutic resistance [26], it is important to know the molecular profiling of each tumor when proposing a treatment plan.

In contrast, *EGFR*-driven tumors (clusters 4 and 5) exhibited distinct co-mutation profiles. NSCLC patients with both mutated *EGFR* and ALK fusions were described in a review by Kemper [27]. In the current study, cluster 4 was characterized by *EGFR* and *ALK* fusions, and demonstrated significantly lower PD-L1, which is in line with a previous publication by Gainor et al. [28]. Moreover, cluster 4 had the lowest TMB levels compared to other clusters. Cluster 4’s composition is indicative of an “oncogene-addicted” phenotype driven by signaling pathways (i.e., *EGFR*) rather than genomic instability. Cluster 5, involving *EGFR* exon 20 insertions, which are relatively rare [29,30], was associated with tumor suppressor loss [31,32]. Cluster 5 patients bearing low TMB are predicted to have shorter survival than those with high TMB (i.e., cluster 2) [33].

Statistically, cluster 4 (*EGFR/ALK*) presented with significantly lower biomarkers (PD-L1, TMB) compared to the *KRAS*-dominant clusters (clusters 1, 2, and 3). Clusters 1, 2, and 3 include mutations *KRAS*, *STK11*, and *TP53*, which may affect the immunogenicity of the patient by increasing PD-L1 expression [34]. *KRAS*, *STK11*, and *TP53* mutations may also affect the presence of accompanying mutations related to genes involved in stabilizing the chromatin (cluster 2) and DNA repairs pathways (cluster 3), as similarly found in pancreatic cancer genomic mutations [35]. This aligns with the hypothesis that *KRAS*, *STK11*, and *TP53* mutations may drive immunogenicity and genomic instability (higher TMB), whereas *EGFR/ALK* alterations (clusters 4 and 5) rely on oncogenic signaling, resulting in “colder” tumors immunologically (with decreased PD-L1 and TMB expression). This means that lung cancer tumors harboring *EGFR/ALK* mutations rely less on genomic instability.

Hence, future research would deepen the investigation on this topic, i.e., as PD-L1 and TMB have clinical significance, and explore the creation of clusters that are in correlation with PD-L1 and TMB. Further studies should assess the effect of immune-therapy-related biomarkers on the clustering in these patients and check the clinical significance by outcomes of the treatments such as efficiency and toxicity.

Frille et al. 2024 [36] presented interactions between four co-mutations in NSCLC: *KRAS*, *STK11*, *TP53* and *KEAP1*. They reported that combined *KRAS*, *G12C* and *TP53* co-occurrence may be targeted by immunotherapy administered to patients diagnosed with advanced tumor stage in stage IV.

The current study does not claim prognostic effects because outcomes were not analyzed. The cluster patterns were contextualized with existing literature that examines *KRAS-STK11-KEAP1* biology in relation to immunotherapy, and *EGFR-TP53* co-mutation as a clinically relevant subgroup in *EGFR*-mutated NSCLC.

Demographically, cluster 4 contained a significantly higher proportion of women and younger patients compared to other clusters, aligning with known epidemiological data regarding *EGFR* mutations [37]. Genetic factors presented in never-smokers were determined in lung cancer arising from oncogenic mutations. Ha et al. studied oncogenic driver mutations in 198 Asian females diagnosed with lung adenocarcinoma [38]. Their analysis of 26 lung-cancer-related genes harboring 214 mutations along with fusion genes found that *EGFR* was the dominant driver gene in 63% of patients. The mutation of *ALK* was detected in 7% of cases. Although cluster 4 consisted of more women than other clusters, it is difficult to draw a clear clinically significant conclusion; it is plausible that the differences were arbitrary or as a result of other variables that were not assessed during the present study.

Chang et al. [39] reported that low-dose computed tomography used for lung cancer screening detected a higher prevalence of subsolid nodules in Asian females than in males. As subsolid nodules may lead to overdiagnosis, it is proposed that this method should be integrated with a multiomics approach. Moreover, Chen et al. [40] suggest that an interdisciplinary strategy should be applied in the clinical setting to optimize precision medicine. Combining factors such as screening protocols and radiologic and genetic data could enhance accuracy, along with polygenic risk scores and molecular biomarkers to refine the stratification of patients.

Further research should be conducted with a bigger sample, and the analysis of clinical results could determine whether there is an effect of these characteristics on the mutation profile in the different clusters.

In addition, *ARID1A* alterations—identified as genomic events—have been investigated in other tumor types as candidate synthetic lethal contexts, including preclinical evidence supporting vulnerability to EZH2 inhibition in *ARID1A*-mutant cells, and broader reviews summarizing multiple synthetic lethal strategies under investigation [41]. These observations are not evidence of efficacy in NSCLC, but support a rationale for documenting *ARID1A* alterations as potential trial-relevant features in future studies.

This study has several limitations: its retrospective design and a modest sample size of 129 patients, as well as heterogeneity in cluster sizes (e.g., *n* = 12 in cluster 2 vs. *n* = 38 in cluster 1), which may affect statistical power. Moreover, the tumor stage was not part of the present study, although different stages (early vs. late stage) may be related to multiple coexisting mutations. Additionally, the lack of behavioral data (smoking status or drinking alcohol) in the database limits the ability to control for environmental confounders while creating a more clinically accurate cluster. The use of an unsupervised algorithm, while mathematically robust, groups purely by proximity without inherent clinical weighting. The pathology repository used for the present study did not contain longitudinal outcome data (e.g., overall survival, progression-free survival, line of therapy, or response assessments) precluding valid hazard ratios/Kaplan–Meier curves in the current dataset. Therefore, the present study analysis was conducted without comparing “driver only” versus “driver + co-mutation” subgroups. This study is also limited by the absence of clinical cofounders (e.g., prior systemic therapy) that may influence mutation patterns.

Future prospective studies are recommended with larger cohorts to validate these clusters and assess their clinical utility regarding treatment efficacy and toxicity. Specifically, investigating the impact of these co-mutation clusters on survival outcomes and resistance mechanisms is warranted.

## 5. Conclusions

In this retrospective cohort, the unsupervised clustering of binary genomic alterations identified reproducible patterns of co-occurring mutations among NSCLC cases with actionable drivers and demonstrated differences in biomarker distribution (PD-L1 and TMB) across clusters. These findings generate testable hypotheses regarding biologically distinct molecular subgroups, but prospective validation in larger cohorts with comprehensive clinical covariates and treatment outcomes is required before clinical prediction claims can be made.

## Data Availability

The raw data supporting the conclusions of this article will be made available by the authors on request.

## Abbreviations

The following abbreviations are used in this manuscript:

MSI	Microsatellite Instability
PD-L1	Programmed Death-Ligand 1
TMB	Tumor Mutational Burden
COPD	Chronic Obstructive Pulmonary Disease
NSCLC	Non-Small Cell Lung Carcinoma
SCLC	Small Cell Lung Carcinoma
ADC	Adenocarcinoma
SCC	Squamous Cell Carcinoma
NGS	Next-Generation Sequencing
LCC	Large Cell Carcinoma
EGFR	Epidermal Growth Factor Receptor
TKI	Tyrosine Kinase Inhibitors
ALK	Anaplastic Lymphoma Kinase
ICI	Immune Checkpoint Inhibitors
CNV	Copy Number Variation
KRAS	Kirsten Rat Sarcoma Virus
STK11	Serine Threonine Kinase 11
TP53	Tumor Protein 53
